# Genome-wide identification, putative functionality and interactions between lncRNAs and miRNAs in *Brassica* species

**DOI:** 10.1038/s41598-018-23334-1

**Published:** 2018-03-21

**Authors:** Jinfang Zhang, Lijuan Wei, Jun Jiang, Annaliese S. Mason, Haojie Li, Cheng Cui, Liang Chai, Benchuan Zheng, Yongqing Zhu, Qing Xia, Liangcai Jiang, Donghui Fu

**Affiliations:** 1 Crop Research Institute, Sichuan Academy of Agricultural Sciences, Chengdu, Sichuan 6110066 China; 2 Chongqing Engineering Research Center for Rapeseed, College of Agronomy and Biotechnology, Southwest University, Chongqing, 400716 China; 3 IFZ Research Centre for Biosystems, Land Use and Nutrition, Justus Liebig University, 35392 Giessen, Germany; 4 Institute of Agro-Products Processing Science and Technology, Sichuan Academy of Agricultural Sciences, Chengdu, Sichuan 6110066 China; 5 Key Laboratory of Crop Physiology, Ecology and Genetic Breeding, Ministry of Education, Agronomy College, Jiangxi Agricultural University, Nanchang, 330045 China

## Abstract

Non-coding RNA (ncRNA) is abundant in plant genomes, but is poorly described with unknown functionality in most species. Using whole genome RNA sequencing, we identified 1885, 1910 and 1299 lncRNAs and 186, 157 and 161 miRNAs at the whole genome level in the three *Brassica* species *B*. *napus*, *B*. *oleracea* and *B*. *rapa*, respectively. The lncRNA sequences were divergent between the three *Brassica* species. One quarter of lncRNAs were located in tandem repeat (TR) region. The expression of both lncRNAs and miRNAs was strongly biased towards the A rather than the C subgenome in *B*. *napus*, unlike mRNA expression. miRNAs in genic regions had higher average expression than miRNAs in non-genic regions in *B*. *napus* and *B*. *oleracea*. We provide a comprehensive reference for the distribution, functionality and interactions of lncRNAs and miRNAs in *Brassica*.

## Introduction

Non-coding RNA (ncRNA) refers to RNAs such as rRNAs, tRNAs and snRNAs that can be transcribed, but which do not encode proteins. Non-coding RNAs play different roles in the cell and in gene expression: common types include ribosomal RNAs (rRNAs) and transfer RNAs (tRNAs), which function in mRNA translation, small nuclear RNAs (snRNAs) involved in splicing, small nucleolar RNAs (snoRNAs) that act to modify rRNAs, and microRNAs (miRNAs) and small interfering RNAs (siRNAs) that regulate the translation and/or stability of mRNA^1^. More recent discoveries include piwi-interacting RNAs (piRNAs), small RNAs which suppress transposon activity and regulate gene expression^2^, and long non-coding RNAs (lncRNAs).

lncRNAs are defined as RNAs more than 200 bp in length but lacking in protein-coding potential^3^. There is accumulating evidence for participation of lncRNAs in a broad range of processes^4^. For example, lncRNAs have been revealed to play major roles in transcriptional regulation, splicing, the organization of nuclear domains and chromatin modification^5,6^. Classification of lncRNAs is a complex task, particularly as functional knowledge is still missing for many identified lncRNAs^4^. Classifications can be based on features such as transcript length, association with annotated protein-coding genes, repeats or other DNA elements of known function, on resemblance to protein coding RNA, on association with a biochemical pathway or subcellular structure, on sequence and structural conservation or on functionality^4^.

LncRNAs have been studied in different tissues and under stress conditions in many plants, and have been shown to play a role in both plant development and various stress responses^7^. Some specific examples include stress-responses in *Arabidopsis*^8^ and in wheat^9^, regulation of photoperiod-sensitive male sterility in hybrid rice^10^, as well as in rice sexual reproduction^11^, where some lncRNAs act as competing endogenous RNAs (ceRNAs). lncRNAs appear poorly conserved between flowering plant species^12–14^, with highly tissue-specific expression^7,12,14^ and gene regulation through either *cis* or *trans* pathways^12,14^. Other genomic elements may also interact with lncRNAs. For example, Wang *et al*. (2015d)^13^ showed that transposable elements play a major role in the origin of *Lycopersicon*-specific lncRNAs in tomato. Wang *et al*. (2015a)^15^ also found lncRNAs functioning as competing endogenous target mimics (eTMs) for microRNAs in tomato response to tomato yellow leaf curl virus (TYLCV) infection.

MicroRNAs (miRNAs) are small noncoding RNAs with the length of 20–24 nt which play a major role in development and various stress responses through silencing of target mRNAs^16^. miRNA genes are transcribed into primary miRNA (pri-miRNA) by polymerase II, and fold into precursor miRNA (pre-miRNA) with a stem-loop structure^17^ before forming into mature miRNA. miRNAs recognize target transcripts through Argonaute (AGO) proteins, which repress mRNAs via binding with 3′ UTR or coding sequences. The degradation of mRNAs by miRNAs occurs through deadenylation, decapping and exonucleolytic digestion^17^. To date, miRNA genes have been identified and characterized in dozens of flowering plant species^18–21^, with ongoing investigations into the role of these small RNAs in regulation of gene expression in many different pathways^22–24^.

*Brassica napus* (AACC; rapeseed) is a young allopolyploid species derived from hybridization of diploid species *B*. *oleracea* (CC; cabbage) and *B*. *rapa* (AA; turnip) <7500 years ago^25,26^. This extremely short evolutionary history makes *B*. *napus* an intriguing model for studies of hybrid and polyploid formation^27^, particularly for rapidly-evolving genomic elements like small RNAs. Recently, the availability of the *B*. *napus*, *B*. *rapa* and *B*. *oleracea* genome sequences^28–30^ has allowed investigation of small RNAs in *Brassica*. A total of 969 miRNAs from 680 miRNA genes have been annotated in *B*. *rapa*, and were found to be more commonly retained than genes during post-polyploidization genome fractionation^31^. As well, 76% of miRNAs were found to be conserved between *B*. *napus* and its progenitor species *B*. *rapa* and *B*. *oleracea*, with recent *MIRNA* expansion and loss events detected in *B*. *napus*^32^. In *B*. *rapa*, 2237 candidate lncRNAs with an average length of 497 bp were identified, and the functions of neighboring genes were analyzed^33^. In addition, a total of 3183 lncRNAs were found to be responsive to pathogen *Sclerotinia sclerotiorum* in *B*. *napus*^34^. However, very little is yet known about function or evolution of lncRNAs in *Brassica*. In this study, we aimed to determine how lncRNAs have evolved and diverged in *B*. *napus* relative to progenitor species *B*. *rapa* and *B*. *oleracea*, and how miRNAs and lncRNAs may functionally interact in each of these species.

## Results

### High-throughput sequencing of *B*. *napus*, *B*. *oleracea* and *B*. *rapa*

To explore the effect of lncRNAs in *Brassica*, lncRNA sequencing of young leaves of *B*. *napus*, *B*. *oleracea* and *B*. *rapa* was performed in two biological replicates. Six strand-specific cDNA libraries were constructed. We removed low quality, adapter and uncertain reads, and obtained a total of 93–102 million clean data reads. About 51–69% of reads mapped to the reference genome (Supplementary Table 1).

In addition, in order to understand the expression of small RNAs in *Brassica*, six small RNA libraries from leaves of *Brassica* species were constructed. After removing low quality, 5′-adapter containing, 3′-adapter null or insert null reads and ploy-N-containing reads, a total of 7.5–10.1 million clean reads were generated. Of these reads, 77–90% were mapped to the genome, of which a further 57–78% mapped to a unique position on the reference genome sequences (Supplementary Table 2). To estimate the reproducibility of the data, correlations between replicated samples were made (Supplementary Figure 1), obtaining correlation coefficients of 0.988, 0.993 and 0.987 for lncRNA sequencing and 0.879, 0.889 and 0.823 for small RNA sequencing in *B*. *napus*, *B*. *oleracea* and *B*. *rapa*, respectively, suggesting high reproducibility between the biological replicates.

### Identification of lncRNAs in *B*. *napus*, *B*. *oleracea* and *B*. *rapa*

**Figure 1:**
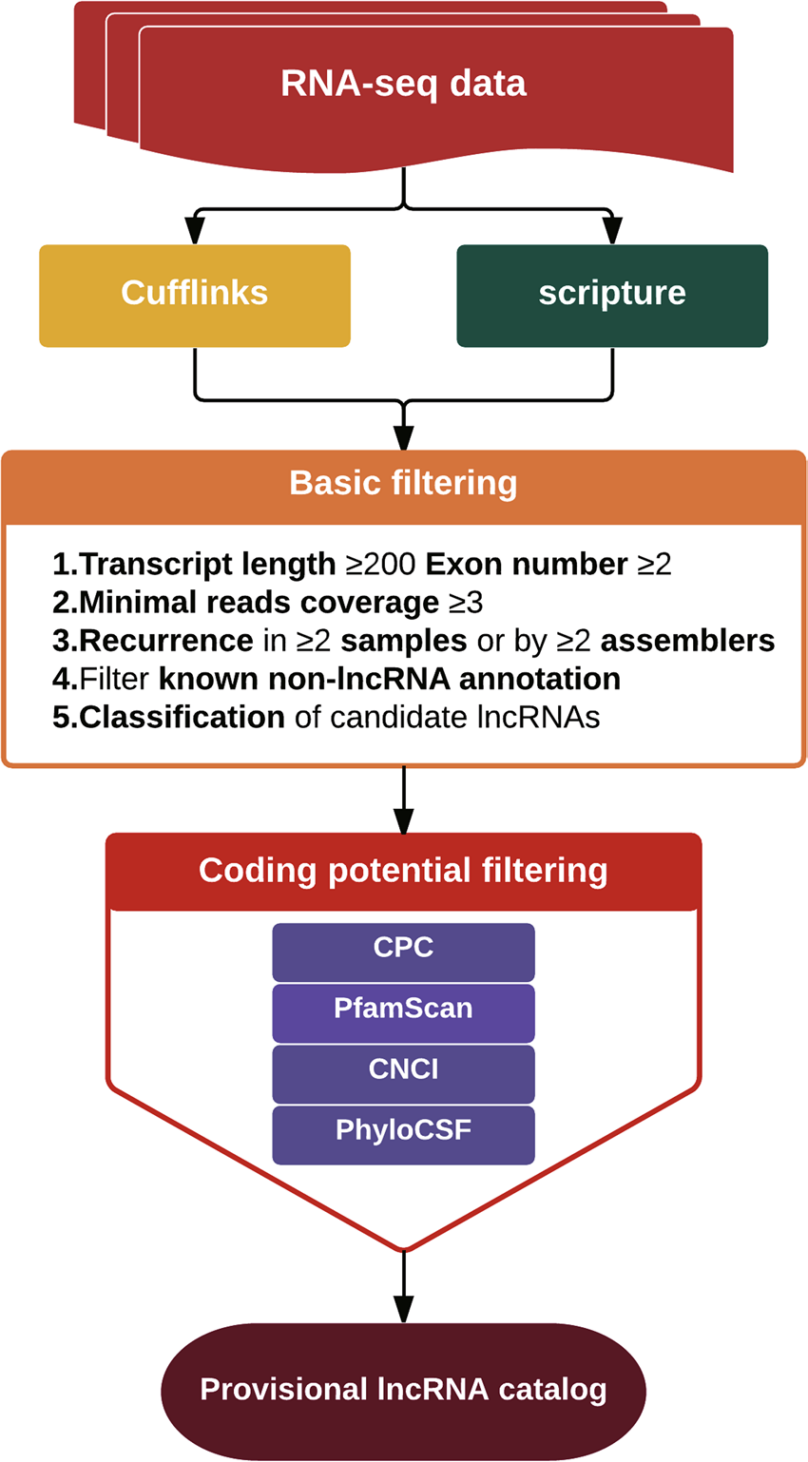
Pipeline for lncRNAs identification from RNA-seq data.

To identify lncRNAs in the three *Brassica* species, we developed a pipeline “RNAseq-*Brassica*” for the RNA-seq data (Fig. 1). After basic filtering and analysis of coding potential, 1885, 1910 and 1299 lncRNAs were found in *B*. *napus*, *B*. *oleracea* and *B*. *rapa*, respectively (Supplementary Table 3). BLASTn analysis was carried out to identify lncRNAs in *B*. *napus* in our study based on all lncRNAs previously published in this species^34^. No previous publications list locations of *B*. *rapa* or *B*. *oleracea* lncRNAs. We found 1483 of 1885 lncRNAs were novel lncRNAs in *B*. *napus*.

**Figure 2:**
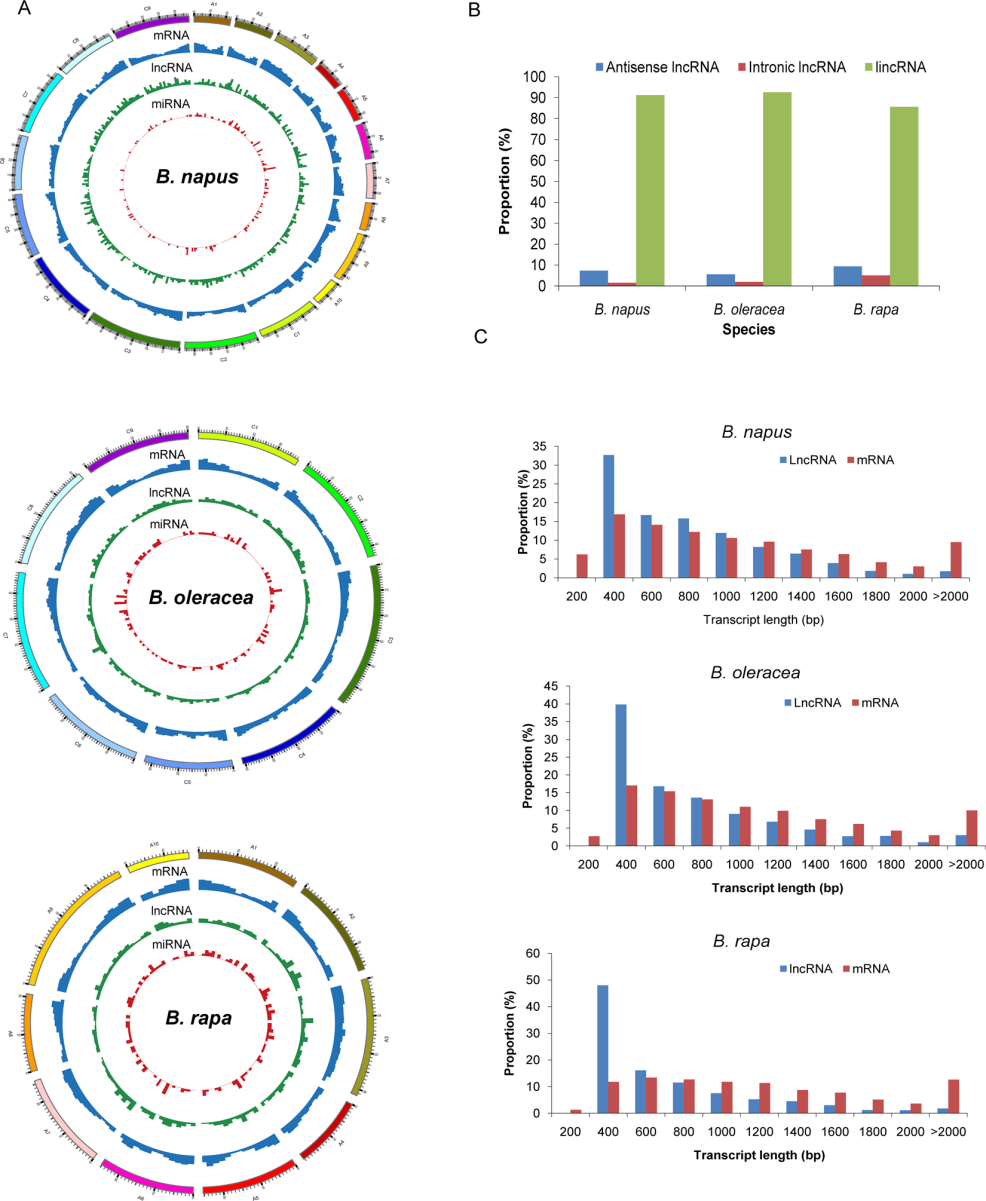
Characteristics of lncRNAs in *B*. *napus*, *B*. *oleracea* and *B*. *rapa*. (**A**) Distribution of mRNAs, lncRNAs and miRNAs on each chromosome. (**B**) Proportion of antisense lncRNAs, intronic lncRNAs and lincRNA identified in *Brassica*. (**C**) Length of lncRNAs in *Brassica* species.

The lengths and distributions of the lncRNAs in the *Brassica* species genomes were analyzed. lncRNAs were evenly distributed across all *Brassica* chromosomes (Fig. 2A). The density of lncRNAs in the *B*. *rapa* and *B*. *oleracea* diploid genomes was 4.17 and 4.31 lncRNAs per Mb, respectively, with 2.00 lncRNAs per Mb in *B*. *napus*. The majority of lncRNAs (91.2%, 92.6% and 85.6%) were located in intergenic regions in *B*. *napus*, *B*. *oleracea* and *B*. *rapa*, respectively (Fig. 2B). The rest of the RNAs were anti-sense lncRNAs (7.27%, 5.50% and 9.39%) and intronic lncRNAs. lncRNA lengths were mostly less than 2 kb, with an average of 1163,1523 and 974 bp in *B*. *napus*, *B*. *oleracea* and *B*. *rapa*, respectively (Fig. 2C). The number of exons in lncRNAs in our study was 4–6 in all three *Brassica* species (although lncRNAs with only one exon were excluded). The structure and organization of lncRNAs was similar across the three *Brassica* species.

### Identification of miRNAs in *B*. *napus*, *B*. *oleracea* and *B*. *rapa*

**Figure 3:**
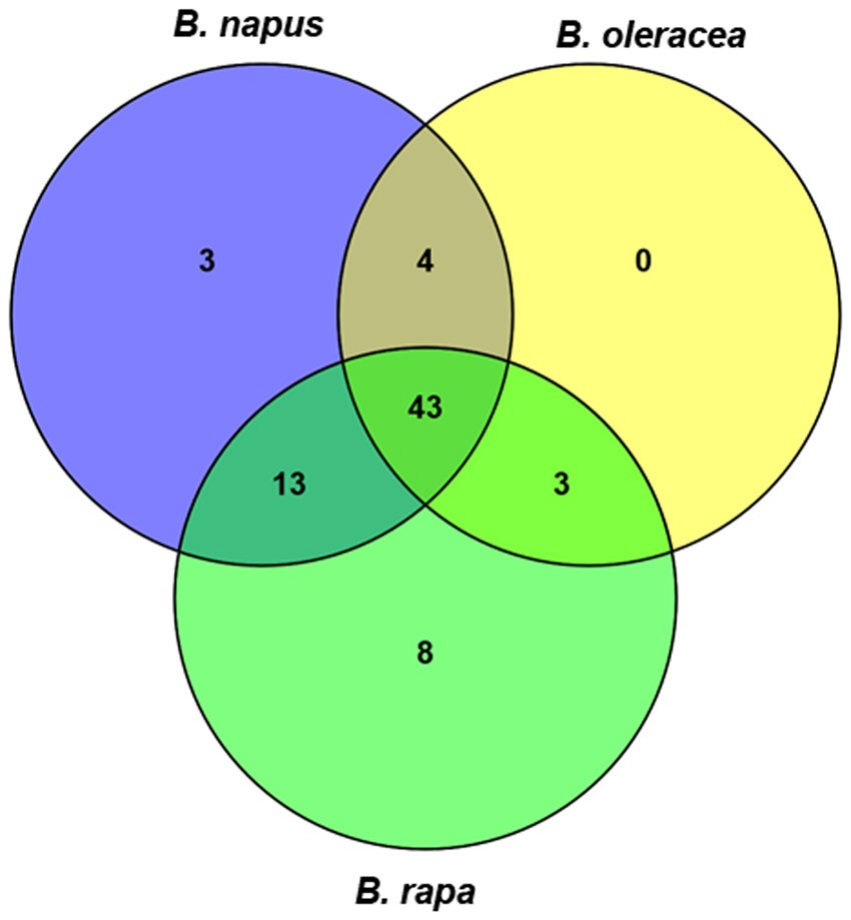
Distribution of conserved miRNA families in *B*. *napus*, *B*. *oleracea* and *B*. *rapa*.

A total of 117, 102 and 123 conserved miRNAs found in *B*. *napus*, *B*. *oleracea* and *B*. *rapa* belonged to 63, 50 and 67 miRNA families, respectively (Supplementary Table 4). Of these 74 miRNA families, 43 miRNA families (75.4%) were present in all three species (such as miR156, miR160, miR162, miR164, miR319), four were present in both *B*. *napus* and *B*. *oleracea* (miR6034, mIR6035, miR9409 and miR9411) and 13 were present in both *B*. *napus* and *B*. *rapa* (Fig. 3). Three families were present in both *B*. *oleracea* and *B*. *rapa* (miR399, miR6036 and miR9410), but absent in *B*. *napus*. In total, three unique miRNA families (miR6028, miR5719 and miR5726) were found in *B*. *napus*, eight in *B*. *rapa* (miR5713, miR5714, miR5716, miR5724, miR9553, miR9556, miR9561 and miR9567) and none in *B*. *oleracea*.

Furthermore, novel miRNAs were discovered with miREvo^35^ and mirdeep 2^36^ according to the hairpin structure of the miRNA precursors, the Dicer cleavage site and predicted free energy. A total of 69, 55 and 38 novel miRNAs were identified in *B*. *napus*, *B*. *oleracea* and *B*. *rapa*, respectively (Supplementary Table 4).

We analyzed the distribution of *MIRNA* (precursor genes of mature miRNA) in the *Brassica* genomes. A total of 168 *MIRNA* were found in *B*. *napus*, of which 32 (20.3%) were located in genes (Supplementary Table 5, Table 1). Of the 32 *MIRNAs* in genes, 12 (37.5%) were within 3′UTRs, seven (21.9%) were within introns, two (6.2%) were within exons, three (9.4%) were in 5′UTRs and eight (25.0%) were located in other genic regions. A total of 144 *MIRNAs* were found in *B*. *oleracea*, of which only five (3.5%) were located in genes, while 14 (9.8%) of the 143 *MIRNAs* in *B*. *rapa* were located in genes. Most *MIRNA* were found in intergenic regions.

**Table 1:** The number of *MIRNAs* located in genes in *Brassica*.

	*B*. *napus*	*B*. *oleracea*	*B*. *rapa*
Intron	7	1	6
3′UTR	12	0	0
5′UTR	3	0	0
Exon	2	1	0
Junctions^a^	8	3	8
Total	32(20.3%)	5(3.5%)	14(9.8%)

^a^Indicates MIRNAs span different genomic regions.

### qRT-PCR validation of lncRNAs and miRNAs

**Figure 4:**
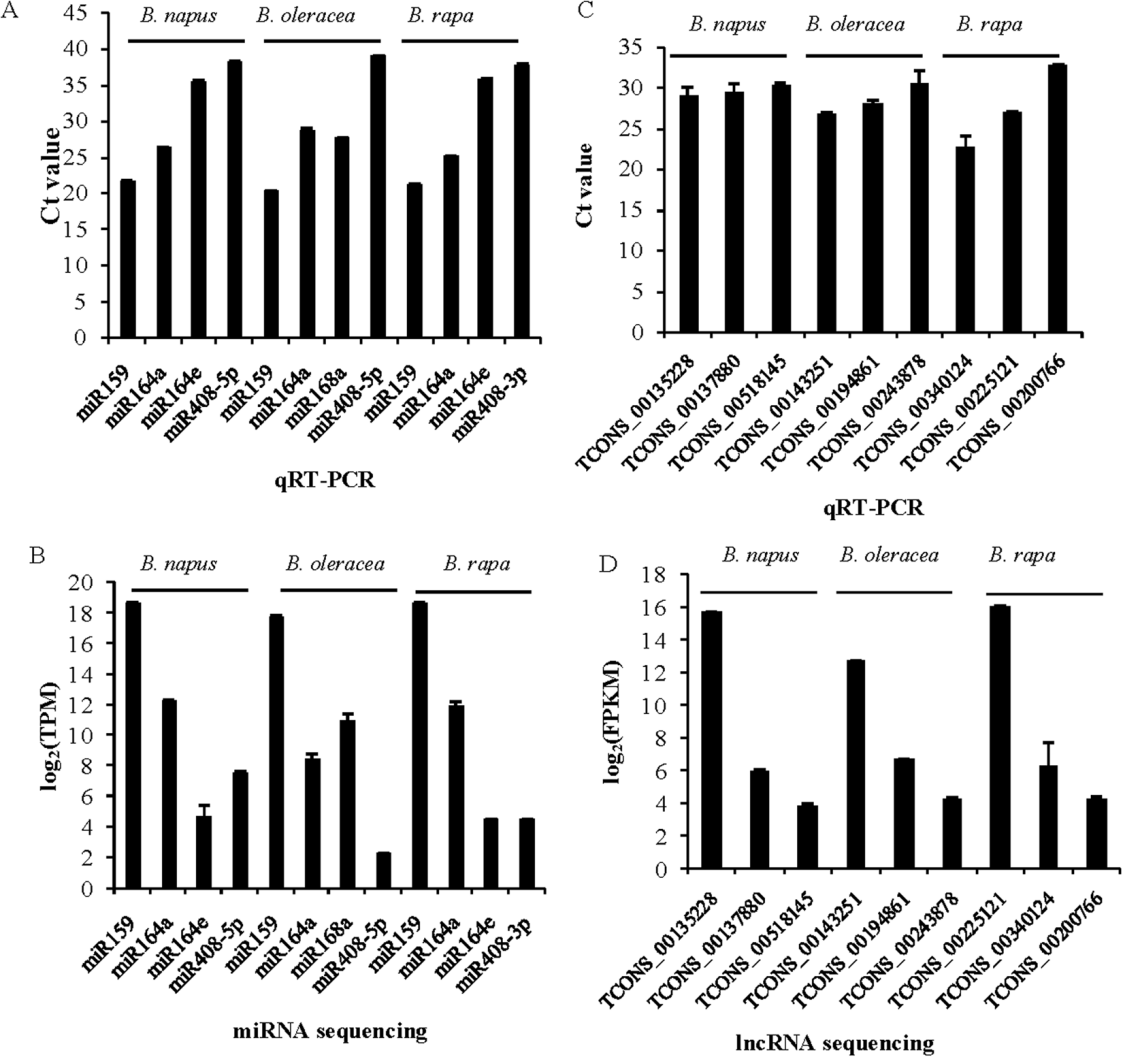
qRT-PCR validation of miRNAs and lncRNAs in *Brassica*.

To validate the expression patterns of lncRNAs in *Brassica*, a total of nine lncRNAs were selected for qRT-PCR analysis (TCONS_00135228, TCONS_00137880 and TCONS_00518145 in *B*. *napus*, TCONS_00143251, TCONS_00194861 and TCONS_00243878 in *B*. *oleracea*, and TCONS_00340124, TCONS_00225121 and TCONS_00200766 in *B*. *rapa*). Expression patterns of all lncRNAs were confirmed to be highly similar between the lncRNA sequencing and qRT-PCR methods (Fig. 4A,B).

In addition, qRT-PCR analyses of 12 miRNAs in the three *Brassica* species were performed to validate the results of miRNA sequencing. Expression patterns for 11/12 miRNAs in the three *Brassica* species were similar between the two methods of miRNA sequencing and qRT-PCR, although miR408-5p in *B*. *napus* had different expression patterns between the two analytical tools (Fig. 4C,D).

### Intermediate conservation of lncRNAs in *Brassica*

We analyzed the homology of lncRNAs in the three *Brassica* species with E < 1e-20 using BLASTn analysis: lncRNAs of *B*. *napus* were used as queries (1885) and lncRNA sequences from *B*. *oleracea* and *B*. *rapa* were used as subjects (Supplementary Table 6). Homologs for 23.1% (435/1885) and 11.0% (208/1885) of lncRNAs were identified in *B*. *oleracea* and *B*. *rapa*, respectively. In addition, lncRNAs of *B*. *oleracea* were used as queries and lncRNA sequences from *B*. *rapa* were used as subjects. In total, 176 of 1910 lncRNAs (9.2%) in *B*. *oleracea* were homologous to lncRNAs in *B*. *rapa* (Supplementary Table 6). A further 1174, 1321 and 916 lncRNAs were found to be specific to *B*. *napus*, *B*. *oleracea* and *B*. *rapa*, respectively. For protein-coding genes, 38,554 of 101,040 genes (38.2%) in *B*. *napus* were homologous to *B*. *oleracea* and 34,255 (33.9%) were homologous to *B*. *rapa*^28^. Therefore, *Brassica* lncRNAs showed intermediate conservation in comparison to protein-coding genes, which is consistent with the conservation of lncRNAs relative to protein-coding genes observed between *A*. *thaliana* and *B*. *rapa* (16%)^37^.

### Repetitive sequences in lncRNAs

Most identified lncRNAs were located in intergenic regions. To understand the origin of these lncRNAs we analyzed repetitive sequences, as repetitive sequences are the major factor driving the emergence of lncNRAs^37,38^. Repetitive sequences include tandem repeats (TRs) that can be classified as satellite, minisatellite and microsatellite repeats, and dispersed repeats that include transposable elements (TEs). We analyzed the locations of lncRNAs in *Brassica* species in relation to repetitive sequences (Supplementary Table 7). In total, 477 of 1885 (25.3%) lncRNAs were found within repetitive sequences in *B*. *napus*, 98.9% of which were located in TR sequences, three of which were located in long terminal repeat (LTR) retrotransposon sequences, and one of which was located within a SINE sequence. In *B*. *oleracea*, 421 of 1901 lncRNAs (22.0%) were located within repetitive sequences, 417 of which (99.0%) were found in TR sequences, and one of which was located in a SINE sequence. In *B*. *rapa*, 304 of 1299 lncRNAs (23.5%) were found within repetitive sequences, of which 243 (79.9%) were located within TR sequences, 67 (22.0%) of which were located in LTR retrotransposon sequences and 9 (2.9%) of which were located in DNA transposons. A total of 1174, 1321 and 916 lncRNAs were found to be specific to *B*. *napus*, *B*. *oleracea* and *B*. *rapa*, respectively. Overall, 278 of 1174 (23.7%), 260 of 1321 (19.7%) and 186 of 916 (20.3%) lncRNAs contained repetitive sequences in *B*. *napus*, *B*. *oleracea* and *B*. *rapa*, respectively. TRs were more likely than other types of TEs to be associated with lncRNAs.

### lncRNAs functioned as precursors or targets of miRNAs

lncRNAs and miRNAs play important roles in the regulation of gene expression. To understand the relationship between lncRNAs and miNRAs, we aligned the precursor sequences of miRNAs to lncRNAs. We found 14 lncRNAs (0.74%) were precursors of 20 miRNAs from 15 miRNA families (10 known and 5 novel miRNA families) in *B*. *napus*, 7 lncRNAs (0.37%) were precursors of 9 miRNAs from 8 miRNA families (4 known and 4 novel miRNA families) in *B*. *oleracea*, and 15 lncRNAs (1.15%) were precursors of 19 miRNAs from 15 miRNA families (10 known and 5 novel miRNA families) in *B*. *rapa* (Table 2). In addition, we found that some conserved miRNAs (miR156, miR159, miR166, miR167, miR168, miR172, miR393, miR1885, miR5654 and miR5718) were produced by lncRNAs in either *B*. *rapa* or *B*. *oleracea* as well as in *B*. *napus*. In addition, lncRNAs were predicted to be the targets of miRNAs in *Brassica*. A total of 18 (0.95%), 26 (1.36%) and 33 (2.54%) lncRNAs were the targets of miRNAs in *B*. *napus*, *B*. *oleracea* and *B*. *rapa* (Table 2). Hence, a fraction of lncRNAs appear to function as either precursors or targets of miRNAs.

**Table 2:** The number of putative miRNA precursors and targets in *Brassica* species.

	*B. napus*	*B. oleracea*	*B. rapa*
Precursors of miRNAs			
Conserved miRNA precursor	9	4	10
Non-conserved miRNA precursor	2	3	2
Common	3	0	3
Total	14(0.74%)	7(0.37%)	15 (1.15%)
Targets of miRNAs			
Conserved miRNA	18	13	27
Non-conserved miRNA	0	11	3
Common	0	2	3
Total	18 (0.95%)	26 (1.36%)	33 (2.54%)

### Expression of lncRNAs

To understand the expression of lncRNAs in *Brassica*, the expression levels (FPKM) were assessed. In all *Brassica* species, mRNAs tended to have higher expression than lncRNAs (Supplementary Figure 2, Table 3). However, some lncRNAs had an expression of more than 15 log_2_(FPKM) in *Brassica*, such as TCONS_00135228 in *B*. *napus* and TCONS_00225121 in *B*. *rapa*, indicating that these lncRNAs may have functional roles, rather than being solely “transcriptional noise”.

**Table 3:** The expression of lncRNAs and miRNAs in *Brassica*.

Expression level	lncRNA log_2_(FPKM)^a^		mRNA log_2_(FPKM)		miRNA log_2_(TPM)^b^	
	Range (Average)	Median value	Range (Average)	Median value	Range (Average)	Median value
An	−4.77~15.69 (7.4)	1.83	−6.68~15.46 (5.6)	3.07	2.05~18.68(12.97)	7.64
Cn	−4.70~14.07 (4.77)	1.74	−7.01~17.07 (5.99)	2.82	2.05~18.68(12.75)	7.49
*B*. *napus*	−4.77~15.69 (6.37)	1.77	−7.01~17.07 (5.97)	2.92	2.05~ 18.68 (12.39)	7.61
*B*. *oleracea*	−5.37~12.74 (3.96)	0.54	−7.76~18.80 (6.49)	2.98	1.29~18.39 (12.64)	6.61
*B*. *rapa*	−4.42~16.02 (7.12)	1.24	−6.65~17.09 (7.12)	3.70	2.51~18.64 (12.60)	7.99

^a^FPKM: fragments per kilobase of exon per million mapped fragments.

^b^TPM: transcripts per million clean tags, normalised using the formula: mapped read count/total reads*1000000.

In addition, we investigated the expression of lncRNAs to determine if expression bias was present between the two subgenomes of *B*. *napus*. The average expression level of lncRNAs in *B*. *napus* was 6.36. The number of lncRNAs in the A subgenome (573) was less than that in the C_n_ subgenome (867), which was consistent with the observation of the two progenitor genomes, where 1299 lncRNAs were identified in *B*. *rapa* (AA genome) and 1910 in *B*. *oleracea* (CC genome) (Table 3). The average expression level (FPKM) of lncRNAs was slightly higher in the A_n_ subgenome (7.40) than in the C_n_ subgenome (4.77) (t-test, *P* > 0.05). In the progenitor species, the average expression of lncRNAs in *B*. *napus* (6.36) and *B*. *rapa* (7.12) was also slightly higher than that in *B*. *oleracea* (3.96) (t-test, *P* > 0.05). However, the expression of mRNAs in the A_n_ subgenome (5.60) and C_n_ subgenome (5.99) was almost the same.

The average expression levels of TR-related, TE-related and unclassified lncRNAs were also analyzed. In *B*. *napus* and *B*. *rapa*, unclassified lncRNAs showed the highest expression levels, 104.88 and 172.82 respectively. In *B*. *oleracea*, TR-related lncRNAs had a slightly higher expression (18.61) than unclassified lncRNA sequences (14.85) (*P* > 0.05). More TE-related lncRNAs were present in *B*. *rapa*, and they showed higher expression levels (25.18) than TR-related lncRNAs (17.19).

### Expression of miRNA

The progenitor species showed similar TPM for miRNA expression: 6369 in *B*. *oleracea* and 6211 in *B*. *rapa*. In *B*. *napus* the average TPM was 5376, which was a little lower than that in the diploid species (Table 3). The average expression level (log_2_TPM) of miRNAs in the A_n_ genome (7.64) was higher than that of miRNAs in the C_n_ genome (7.49) (t-test, *P* > 0.05) in *B*. *napus*. This was consistent with the expression of lncRNAs in the subgenomes of *B*. *napus*.

**Figure 5:**
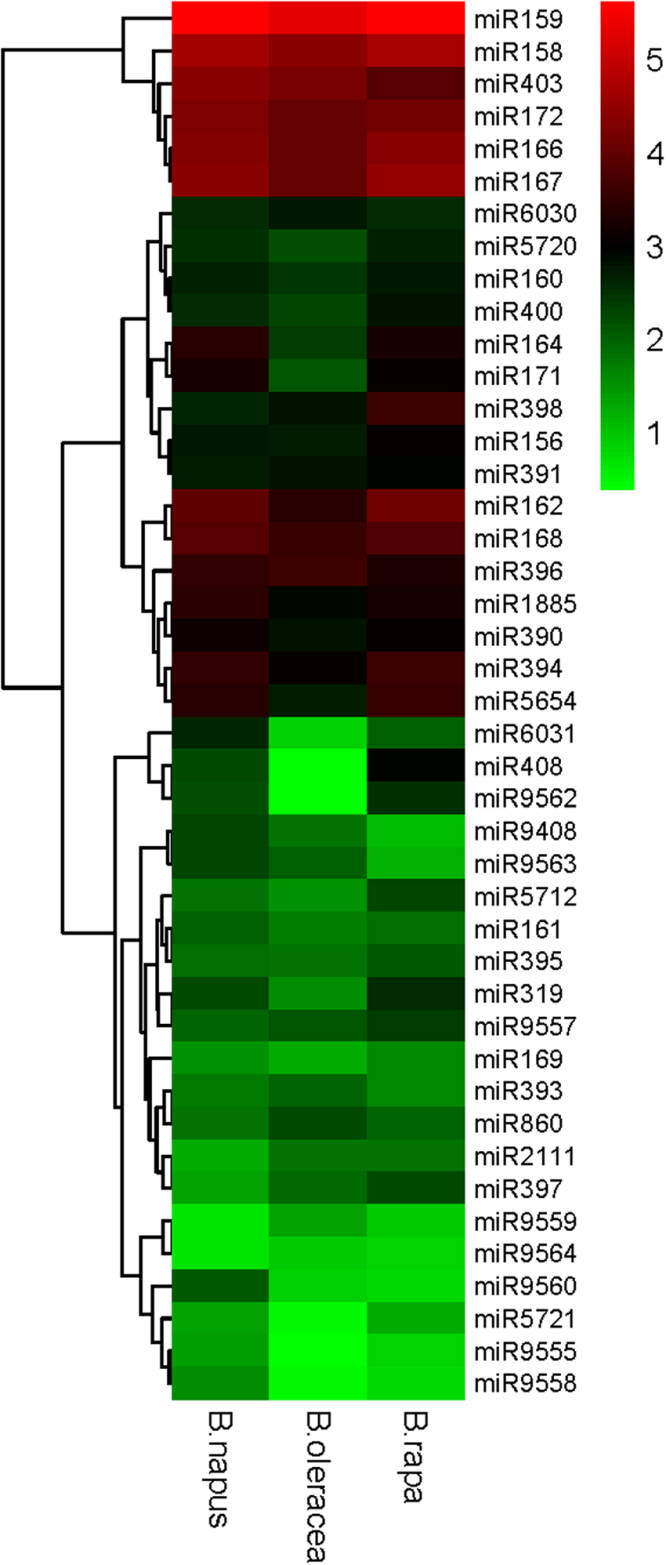
Expression of conserved miRNA families in *Brassica*. The expression levels are given in log_10_(TPM). TPM, transcripts per million clean tags.

In addition, miRNAs in genic regions had higher average expression than miRNAs in non-genic regions in *B*. *napus* and *B*. *oleracea*, but this finding was solely related to the position of the *MIRNA*159 gene in *Brassica* species. When miRNA159 was excluded from the analysis, the expression of miRNAs in non-genic regions was higher than that of miRNAs in genic regions in *Brassica*. The expression level of conserved miR159 was the highest of any miRNA in all three species: 420561 in *B*. *napus* (located in a genic region), 212657 in *B*. *oleracea* (located in a genic region) and 407567 *B*. *rapa* (located in a non-genic region), respectively (Fig. 5). The predicted target genes of miR159 mainly encoded MYB TFs, such as MYB81, MYB101, MYB65, MYB97, MYB120 and sporocyteless (SPL).

The expression levels of 43 common conserved miRNAs were also compared in *Brassica*, and found to average 13830 in *B*. *napus*, 7113 in *B*. *olerace*a and 13665 in *B*. *rapa*. Differentially expressed families were identified based on the criteria log_2_(fold change) > 2. A total of 12 miRNA families showed differential expression between *B*. *napus* and *B*. *oleracea*, eight between *B*. *napus* and *B*. *rapa*, and 13 between *B*. *oleracea* and *B*. *rapa*. The expression of miR9558 and miR9560 was up-regulated in *B*. *napus* relative to in *B*. *rapa* and *B*. *oleracea* (Fig. 5). Only the target genes of miR9558 were found, and these mainly participated in DNA synthesis and post-translational modification.

### Target gene identification for miRNAs

To understand the role of miRNAs in *Brassica* gene regulation, target genes were predicted by psRobot^39^. A total of 61 miRNA families were targeted to 1080 genes in *B*. *napus*, 45 miRNA families were targeted to 340 genes in *B*. *oleracea* and 67 miRNA families were targeted to 906 genes in *B*. *rapa* (Supplementary Table 8). Some of the potential targets were transcription factors, and some were genes related to biotic and abiotic stress responses. A total of 161 genes were orthologous between *B*. *rapa* and *B*. *oleracea*, while 356 genes (33.0%) in *B*. *rapa* and 241 genes (71.2%) in *B*. *oleracea* were orthologous to those in *B*. *napus*. Gene functions were also similar in the three *Brassica* species. GO enrichment analysis showed that the target genes of miRNAs in *Brassica* were mainly enriched in cell death, multicellular organismal processes, developmental processes, defense response and immune system processes (Supplementary Table 9).

## Discussion

We analyzed lncRNAs and miRNAs at the whole genome level in *B*. *napus* and its two progenitor species, *B*. *oleracea* and *B*. *rapa*. Very few studies to date have analysed lncRNAs in *Brassica*: one study previously identified 2237 lncRNAs in *B*. *rapa*^33^, while another identified 3183 lncRNAs in *B*. *napus* expressed in response to *Sclerotinia*^34^. In the present study we identified a total of 1885, 1910 and 1299 lncRNAs in *B*. *napus*, *B*. *oleracea* and *B*. *rapa*, respectively, significantly adding to the number of lncRNAs identified in *Brassica* species. Most lncRNAs (more than 85.6%) were located in intergenic regions in our study, consistent with previous studies in other species^14,15^. The density of lncRNAs was much lower in *B*. *napus* (2.00 lncRNAs per Mb) relative to its diploid progenitors *B*. *rapa* (4.17 lncRNAs per Mb) and *B*. *oleracea* (4.31 lncRNAs per Mb): this finding supports previously identified dynamics of “genome downsizing” via loss of repetitive elements and high-copy number sequences as a result of allopolyploid formation^40,41^.

A total of 117, 102 and 123 conserved miRNAs belonging to 63, 50 and 67 miRNA families were found in *B*. *napus*, *B*. *oleracea* and *B*. *rapa* respectively, including 69, 55 and 38 novel miRNAs. Most *MIRNAs* (80–90%) were located in intergenic regions. In addition, many more miRNAs (20.3%) were located in genes in *B*. *napus* than in *B*. *rapa* (3.5%) or in *B*. *oleracea* (9.8%), suggesting that these miRNAs may assist in the more complex gene regulation required in *B*. *napus* post-polyploidization. This finding supports previous work suggesting that miRNAs played an important role in polyploidization and subsequent genome evolution in *B*. *napus*^32,42^.

In our study, we found that the expression of lncRNAs and miRNAs in the A_n_ subgenome was higher than in the C_n_ subgenome in *B*. *napus*. The average expression level of A_n_ genome lncRNAs was also higher than the average expression level of C_n_ genome lncRNAs, and higher in A genome species *B*. *rapa* than in C genome species *B*. *oleracea*. The same trend of higher expression in the A genome relative to the C genome was found for miRNA (*P* > 0.05), as well as a higher abundance of miRNA reads. This is consistent with previous results identifying biased subgenome expression of miRNA and lncRNAs in *B*. *napus* and cotton, respectively^32,43,44^. This result supports the putative genome-wide homoeolog expression level bias^45^ between the A and the C genomes. The C subgenome of *B*. *napus* tends to be more readily lost than the A genome^28,46^, and is putatively more heavily silenced due to its increased burden of repetitive sequences relative to the A genome^30^; the A genome is therefore proposed to be “dominant” to the C genome in overall gene expression.

In general, miRNAs are moderately conserved between plant species^32,47^. Our study supported this result, with the identification of 43 of 74 conserved miRNA families (75.4%) present in all three *Brassica* species. In addition, we found some miRNAs that were putatively newly generated or lost after polyploidization: target genes for these miRNAs in *B*. *napus* were related to cell wall proteins and stress response, possible candidates for species adaptation processes. In contrast to miRNAs, lncRNAs showed poor conservation, with only 23 homologous lncRNAs out of the >5000 identified in the three *Brassica* species. This finding is consistent with previous studies, which support lncRNAs diversity and rapid evolution in plant species^13,48^. Although lncRNAs show rapid evolution, some conservation of function is predicted. lncRNA has previously been shown to be involved in plant developmental processes: *FLC* (*FLOWERING LOCUS C*) regulates flowering time in *A*. *thaliana*, and three lncRNAs, COOLAIR (CILD INDUCED LONG ANTISENSE INTRAGENCI RNAs), COLDAIR (COLD ASSISTED INTRONIC NONCODING RNA) and ASL (Antisense Long), modify FLC through epigenetic regulation^5,49,50^. lncRNAs may also be involved in phosphate (essential for plant growth and development) homeostasis in *A*. *thaliana* and rice^51,52^, and were also associated with putative phosphate metabolic process genes in our study. In addition, lncRNAs play a major role in various stress responses^7,15^, which might contribute to environmental adaptation in speciation. Wang *et al*. (2015c)^53^ identified lncRNAs expressed under osmotic and salt stress conditions in *Medicago truncatula* which were likely involved in adaptation to abiotic stresses.

Transcription factors were the main putative target genes of miRNAs in our study. Several of these transcription factors could be important in speciation. MYB101 functions in pollen tube reception in *A*. *thaliana*^54^, while ARF6 and ARF8 promote flower maturation in *A*. *thaliana*^55^. In addition, resistance genes accounted for a high proportion of genes associated with miRNAs in our study. Disease resistance genes have also been implicated in speciation via reproductive isolation, due to their potential relationship to hybrid necrosis^56,57^, and may contribute to differential environmental adaptation in newly formed species. However, a great deal more research still needs to be done to fully elucidate the evolutionary and regulatory functionality of miRNAs and particularly lncRNAs in the *Brassica* genus. Our research offers a tantalizing glimpse at possibilities for how these two classes of small RNAs may interact in polyploidization and speciation processes.

## Materials and Methods

### lncRNA sequencing and small RNA sequencing

Young leaves from single accessions of *B*. *rapa*, *B*. *oleracea* and *B*. *napus* (five lines per accession) were collected, pooled together and immediately frozen in liquid nitrogen for lncRNA sequencing and small RNA sequencing, with two biological replicates per accession. The *B*. *oleracea* (kale) sample was a DH line generated from accession “15M2143”, with black seeds and a long growth period. Semi-winter *B*. *rapa* “Yaanhuangyoucai” was a sixth generation self-pollinated inbred line from a local variety in Sichuan Agricultural University at Ya’an (187–190 day growth period and 30 day flowering period, 87% yellow-seeded/13% brown-seeded). Semi-winter brown-seeded *B*. *napus* “G184–189” was an eighth-generation self-pollinated inbred selection from the Sichuan Academy of Agricultural Sciences (226–229 day growth period, 30 day flowering period).

For lncRNA sequencing, ribosomal RNA was removed by an Epicentre Ribo-zerp^TM^ rRNA Removal Kit (Epicentre, USA). Subsequently, sequencing libraries were generated using the rRNA-depleted RNA by NEBNext® Ultra™ Directional RNA Library Prep Kit for Illumina® (NEB, USA) following the manufacturer’s recommendations. The libraries were sequenced on an Illumina Hiseq2000 platform and 100 bp paired-end reads were generated.

Small RNA sequencing libraries were generated using NEBNext® Multiplex Small RNA Library Prep Set for Illumina® (NEB, USA.) following manufacturer’s recommendations, and index codes were added to attribute sequences to each sample. The libraries were sequenced on an Illumina Hiseq2500/2000 platform and 50 bp single-end reads were generated.

### Identification of lncRNAs

Indices of the reference genomes of *B*. *napus*, *B*. *oleracea* and *B*. *rapa*^25,29,58^ were built using Bowtie v2.0.6 (Broad Institute, Cambridge, MA, USA)^59^. Sequencing reads were aligned to the reference genome using TopHat v2.0.9^60^, and assembled by both Scripture (beta2)^61^ and cufflinks (v2.1.1)^62^.

We selected transcripts which met the following criteria: length ≥ 200 bp; read coverage > 3; presence in both sample replicates and both assemblies (Cufflinks and scripture). We then filtered for known non-lncRNA annotation and classified remaining transcripts as candidate lncRNAs. We subsequently performed coding potential filtering using Coding Potential Calculator (CPC) and Pfam-scan. CPC (Coding Potential Calculator) (0.9-r2) assesses the extent and quality of the open reading frame (ORF) in a transcript, and attempts to match sequences with a known protein sequence database to classify transcripts as coding vs. non-coding. We used the NCBI eukaryote protein database and set the e-value cut-off to 1e^−10^ in our analysis^63^. Pfam Scan (v1.3) was used to identify occurrences of any of the known protein family domains documented in the Pfam database (release 27; used both Pfam A and Pfam B)^64^. Any transcript with a Pfam hit was excluded from the following analysis steps. Pfam searches used default parameters^65^.

### Identification of known and novel miRNA

Small RNA tags were mapped to the reference genome by Bowtie^59^ without permitting mismatches. We then removed the tags originating from protein-coding genes, repeat sequences, rRNA, tRNA, snRNA, snoRNA and other small RNA tags. miRBase20.0 was used as the reference database for known miRNA. Modified software “mirdeep2”^36^ and “srna-tools-cli” were used to obtain the potential miRNAs and draw the predicted secondary structures.

The hairpin structure of miRNA precursors can be used to predict novel miRNAs. Novel miRNAs were identified with “miREvo”^35^ and “mirdeep2”^36^ through secondary structure, minimum free energy and Dicer cleavage site characteristics. Briefly, the secondary structures of miRNA precursors were detected using Mfold^66^, and the structure with the minimum free energy was selected^67^.

### Characterization of lncRNAs and miRNAs

The distribution of lncRNA and *MIRNA* genes in the genome was visualized using Circos^68^. To evaluate the lncRNAs that may act as precursors of miRNAs, we aligned the lncRNAs with identified miRNAs (e-value = 1e-5). lncRNAs as targets of miRNAs were predicted by psRNATarget (http://plantgrn.noble.org/psRNATarget/) with default values^69^. The rate of non-synonymous substitutions (Ka) and the rate of synonymous substitutions (Ks) of coding genes were determined by PAML-condem^70^.

### Expression of lncRNAs and miRNAs

The expression levels of lncRNAs and coding genes were estimated using FPKM (fragments per kilobase of exon per million mapped fragments). The expression of miRNAs was normalized to TPM (transcripts per million clean tags)^71^ using the formula: TPM = mapped read count/total reads*1000000. Differentially expressed miRNAs between species were identified based on log_2_(fold change) ≥ 2.

### qRT-PCR

To validate the results of miRNA and lncRNA sequencing, qRT-PCR was conducted in the three *Brassica* species. Primers are listed in Supplementary Table 10 (RiboBio Co.). PCR reactions contained 10 μL SSoAdvanced SYBR Green Supermix (Bio-Rad), 2.0 μL cDNA, 1 µL primer, and distilled water to a final volume of 20 µL. Two independent biological replicates, each with three technical replicates, were run for test genes. The cycle threshold (Ct) was determined using the default settings.

## Electronic supplementary material

Supplementary Figures

Dataset 1
