# Supplementary material for: Genome-wide identification, putative functionality and interactions between lncRNAs and miRNAs in Brassica species
Source: Sci Rep. 2018 Mar 21;8:4960. doi: 10.1038/s41598-018-23334-1 (PMC5862966; doi:10.1038/s41598-018-23334-1)
Supplement: Supplementary file 1 — Supplementary Figures [file 41598_2018_23334_MOESM1_ESM.pdf]

Genome-wide identification, putative functionality and interactions between lncRNAs and miRNAs in *Brassica* species

Jinfang Zhang[1,*], Lijuan Wei[2,*], Jun Jiang[1], Annaliese S. Mason[3], Haojie Li[1], Cheng Cui[1], Liang Chai[1], Benchuan Zheng[1], Yongqing Zhu[4], Qing Xia[1], Liangcai Jiang[1,6], Donghui Fu[5,6]

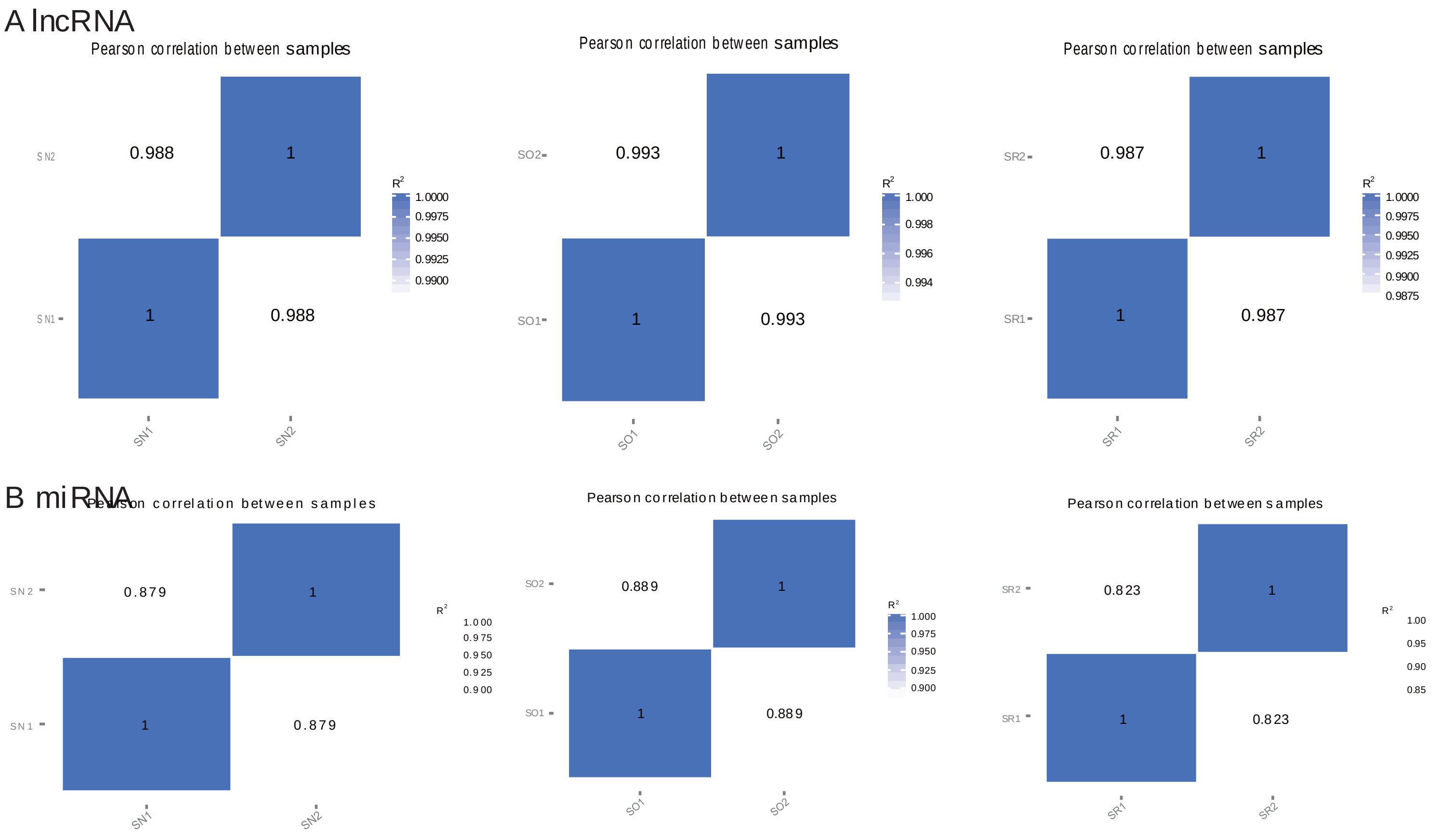

Supplementary Figure 1 The correlation of lncRNA (A) and miRNA (B) between replicated samples in *B. napus*, *B. oleracea* and *B. rapa*. SN1 and SN2: replicated samples of *B. napus*. SO1 and SO2: replicated samples of *B. oleracea*. SR1 and SR2: replicated samples of *B. rapa*.

Genome-wide identification, putative functionality and interactions between lncRNAs and miRNAs in *Brassica* species

Jinfang Zhang[1,*], Lijuan Wei[2, *], Jun Jiang[1], Annaliese S. Mason[3], Haojie Li[1], Cheng Cui[1], Liang Chai[1], Benchuan Zheng[1], Yongqing Zhu[4], Qing Xia[1], Liangcai Jiang[1,6], Donghui Fu[5,6]

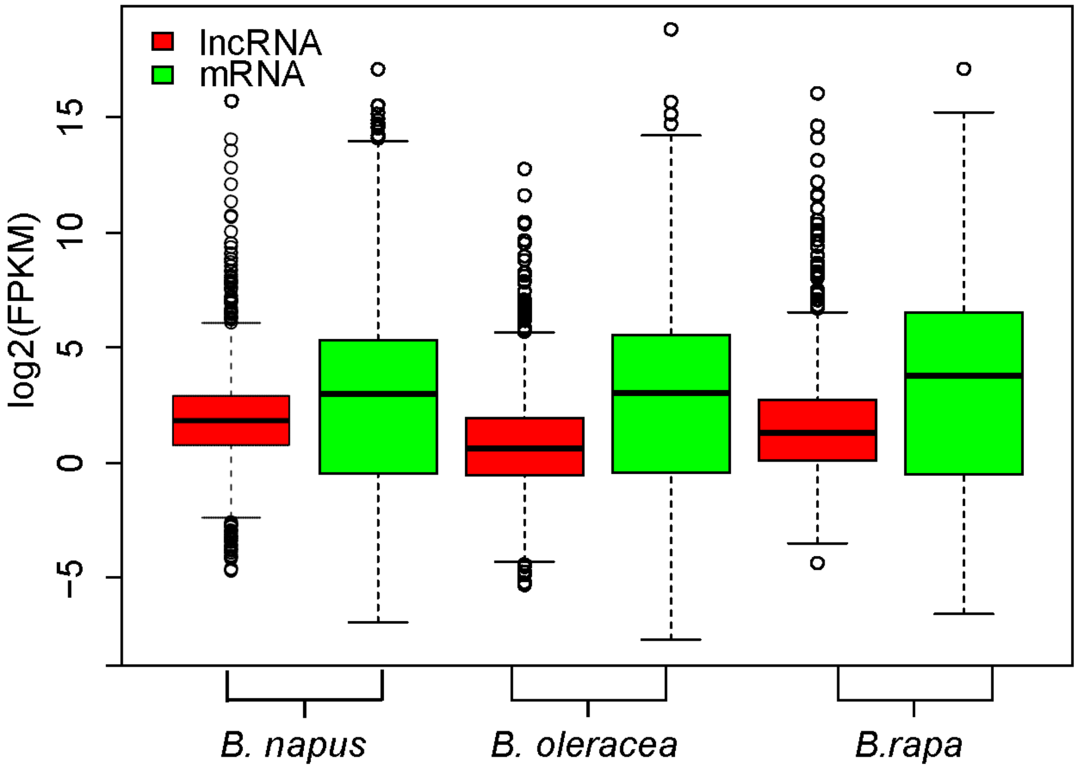

Supplementary Figure 2 Expression of lncRNA and miRNA in *Brassica*
